# Heterotrophic Compensation: A Possible Mechanism for Resilience of Coral Reefs to Global Warming or a Sign of Prolonged Stress?

**DOI:** 10.1371/journal.pone.0081172

**Published:** 2013-11-21

**Authors:** Adam D. Hughes, Andréa G. Grottoli

**Affiliations:** 1 Scottish Association for Marine Science, Oban, Scotland, United Kingdom; 2 The School of Earth Sciences, The Ohio State University, Columbus, Ohio, United States of America; University of Hong Kong, Hong Kong

## Abstract

Thermally induced bleaching has caused a global decline in corals and the frequency of such bleaching events will increase. Thermal bleaching severely disrupts the trophic behaviour of the coral holobiont, reducing the photosynthetically derived energy available to the coral host. In the short term this reduction in energy transfer from endosymbiotic algae results in an energy deficit for the coral host. If the bleaching event is short-lived then the coral may survive this energy deficit by depleting its lipid reserves, or by increasing heterotrophic energy acquisition. We show for the first time that the coral animal is capable of increasing the amount of heterotrophic carbon incorporated into its tissues for almost a year following bleaching. This prolonged heterotrophic compensation could be a sign of resilience or prolonged stress. If the heterotrophic compensation is in fact an acclimatization response, then this physiological response could act as a buffer from future bleaching by providing sufficient heterotrophic energy to compensate for photoautotrophic energy losses during bleaching, and potentially minimizing the effect of subsequent elevated temperature stresses. However, if the elevated incorporation of zooplankton is a sign that the effects of bleaching continue to be stressful on the holobiont, even after 11 months of recovery, then this physiological response would indicate that complete coral recovery requires more than 11 months to achieve. If coral bleaching becomes an annual global phenomenon by mid-century, then present temporal refugia will not be sufficient to allow coral colonies to recover between bleaching events and coral reefs will become increasingly less resilient to future climate change. If, however, increasing their sequestration of zooplankton-derived nutrition into their tissues over prolonged periods of time is a compensating mechanism, the impacts of annual bleaching may be reduced. Thus, some coral species may be better equipped to face repeated bleaching stress than previously thought.

## Introduction

Coral reefs are of critical ecological, economic, and cultural importance, providing ecosystem services with an estimated value of hundreds of billions of dollars annually [1]. Reef building corals exhibit mixotrophy, relying on both the photoautotrophic products of their endosymbiotic algae and the nutrients acquired through heterotrophic predation [2]. This mixotrophy results in a complex cycling of inorganic and organic carbon between the coral host, the skeleton it secretes, and its endosymbiotic algae [3,4]. However, during thermal bleaching caused by elevated seawater temperatures the coral-algae relationship breaks down and there is a dramatic reduction in the concentration of endosymbiotic algae [5-7] and/or the endosymbiotic algal pigments [8,9]. This results in a substantial reduction in the assimilation of photoautotrophically derived organic carbon [4]. 

At an ecosystem level these thermally induced events can result in mass coral bleaching events where over 90% of the coral in any one area become bleached, often leading to significant coral mortality [10]. The occurrence of mass bleaching events is predicted to increase in frequency [11] and threatens to reduce reefs globally by 60% [12]. However not all bleaching events will result in the mortality of the coral colony; some corals will bleach and recover, whilst others might not visibly bleach at all [13-15]. For the surviving coral colonies the period between successive bleaching events allows the opportunity to recover from the physiological impacts of the bleaching event, acting as a temporal refugium analogous to a spatial refugia [16]. Predicting the response of coral reefs to repeated bleaching events is dependent on both defining the size of this temporal refuge, and on understanding any adaptive strategies that the coral holobiont may employ to recover within the limits of the temporal refuge or to increase the size of this temporal refugia. One such adaptive strategy is the ability of the coral animal to host multiple clades of endosymbiotic algae [17] and that a switch to more thermo-tolerant clades of endosymbiotic algae increases the resistance of recovering reefs to future bleaching [18]. This increases the size of the temporal refuge. If the recovery period is greater than the temporal refugia, then bleaching is likely to occur before the coral has fully recovered, thus lowering the resilience of the coral to that bleaching event. Prolonged elevated levels of heterotrophy may present another adaptive strategy for increasing the resistance of corals to bleaching and in hastening recovery from bleaching.

During a bleaching event photosynthetic rates of the holobiont may be reduced by up to 90% [9,19,20] radically reducing the energy available to the coral holobiont. In some species, thermal bleaching triggers a switch to increased heterotrophic feeding [21], and this trophic switching is an important determinant of colony survival after bleaching [22]. It has long been known that some species of reef corals can survive for long periods without sunlight [23]. Heterotrophically acquired carbon is important in tissue building in corals and anemones [4,24] and can reduce the severity of bleaching [25]. However the proportionate contribution of heterotrophy and photoautotrophy to the coral diet during long-term recovery from thermal bleaching and the importance of either pathway during this process is poorly understood. This study aims to understand the role that these respective pathways play in the recovery of corals over the course of almost a year following thermal bleaching. Using ^13^C enriched dissolved inorganic carbon (DIC) in seawater to label the photoautotrophic pathways and ^13^C enriched rotifers to label the heterotrophic pathway, the proportionate contribution of both sources of carbon was assessed in two species of Hawaiian coral for 11 months following an experimental bleaching. 

## Materials and Methods

Coral specimens were collected at 2-4m depth from the fringing reef surrounding Moku O Lo‘e Island at the Hawaii Institute of Marine Biology in Kaneohe Bay, Hawaii on 11 August 2006. Five large healthy colonies of *M. capitata* (branching type) and *P. compressa* were identified from which 5 cm tall coral branch tip fragments were collected. This was performed under special activity permits SAP 2007-28 and SAP 2008-4 issued by the Hawaii Department of Land and Natural Resources. This study was carried out in strict accordance with the regulations and recommendations of the Ohio State University for the care of and use of animals. The fragments were attached to 20x20mm ceramic tiles and placed in 16 flow-through seawater tanks and allowed to acclimatize for 7 days. In total 54 fragments from each of 5 colonies were collected. 

**Figure 1:**
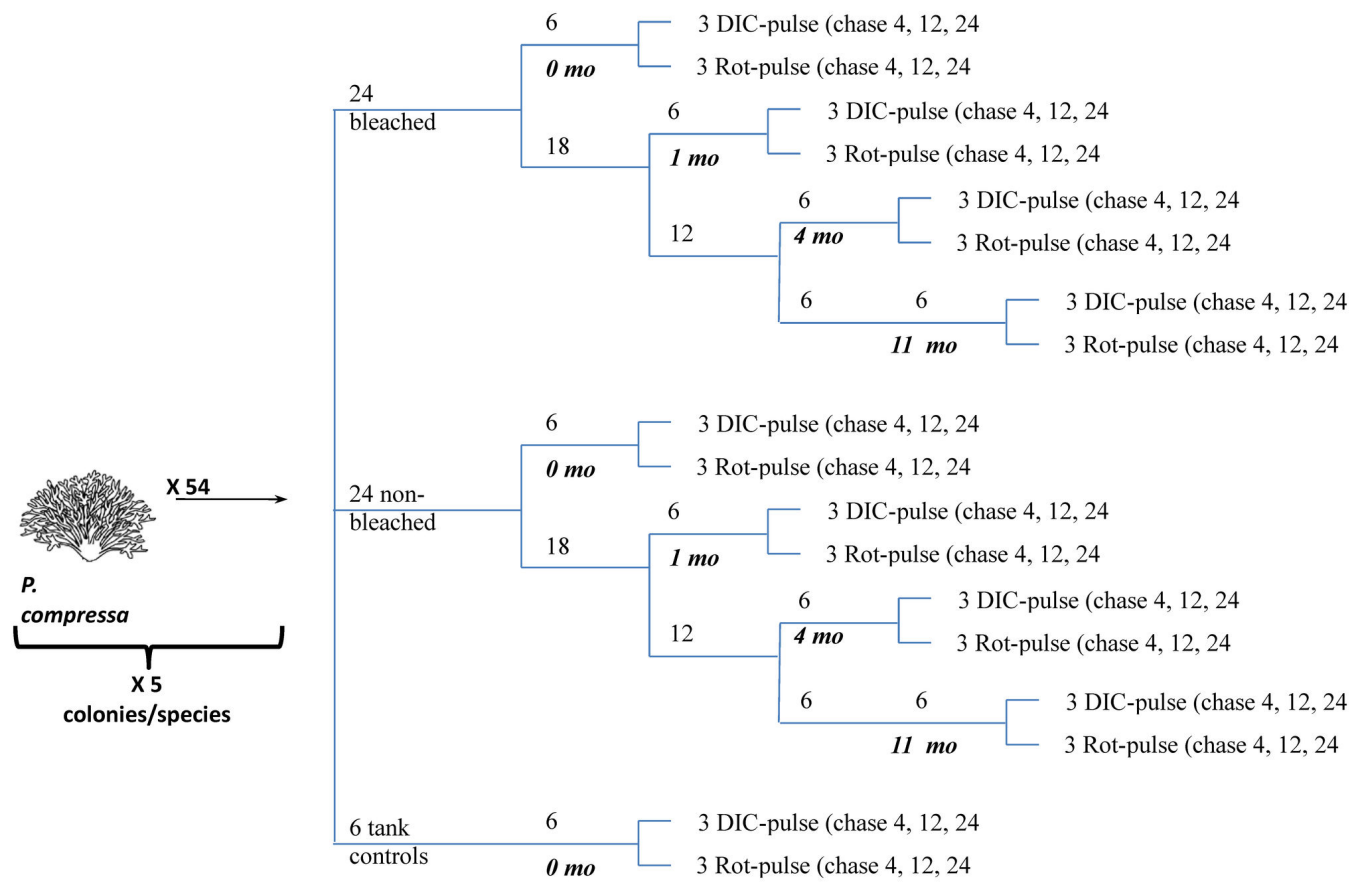
Flow diagram of experimental method. This method was used for both *Porites compressa* and *Montipora capitata* corals. Mo rec = months of recovery, DIC-pulse = 13C-labeled dissolved inorganic carbon, Rot-pulse = 13C-labeled rotifers, hrs = hours.

Following acclimatization the fragments from each colony were divided into 2 sets of 24 fragments and one set of 6 fragments. One set of 24 fragments from each colony was bleached by exposing them to elevated seawater temperatures for three weeks (Figure 1). A second set of 24 fragments from each colony was kept in ambient seawater as non-bleached controls (Figure 1). The last set of 6 fragments from each colony was returned to the reef as tank controls for the same three weeks. Following these three weeks, 18 fragments per colony from each of the bleached and non-bleached control sets were returned to the reef to recover in situ. 

The 6 remaining fragments from each of the bleached, non-bleached control, and tank control sets, were pulse-labelled in tanks as follows. Three fragments from each colony within each set of corals (i.e., bleached, non-bleached controls, tank controls) were pulse-labelled through their photoautotrophic pathway by incubating them in ^13^C-labelled bicarbonate (HCO_3_^-^) in seawater for 8 hours during the day (average δ^13^CVPDB=799‰) then returning them to flow-through seawater tanks and one fragment per colony was collected after a 4-, 12-, and 24-hours chase period. An additional 3 fragments from each colony within each set of corals were labelled through their heterotrophic pathway by feeding them^13^C-labeled rotifers (average δ^13^CVPDB=6216‰) for 11 hours at night, then returned to flow-through seawater tanks and one fragment from each colony was collected after 4-, 12-, and 24-hours chase period. Figure 1 shows a schematic of the experimental procedure. 

All collected fragments were immediately frozen and returned to the lab. Tissue was removed from the skeleton using an airbrush and each fragment was separated into its animal host and endosymbiotic algae through centrifugation as described in Hughes et al (2010) [4] . The δ^13^C of each component was measured and the δ^13^C enrichment relative to natural abundance δ^13^C values reported in [26] was calculated. The variation in natural abundance ranges by 2-5‰VPBD [27,28] which is very small compared to the level of enrichment measured in this experiment (20-100‰VPBD). Thus any natural variation in δ^13^C is insignificant relative to the level of enrichment. A complete description of the coral bleaching method, labelling methods, sample preparation, and isotopic analyses for the corals immediately after bleaching is detailed in Hughes et al. (2010) [4]. Except for the reef controls, this process was repeated after one, four and 11 months of recovery. 

### Statistical Analysis

Differences in the levels of tissue enrichment at each recovery interval and for each species and stable isotope label source was tested using a two way Analysis of Variance (ANOVA), where treatment (bleached, non-bleached control) and tissue (coral host, skeleton, endosymbiotic algae) were the factors. Data from the three chase periods (4,12,24) were used as separate replicates. The model for the ANOVA was as follows: X = µ + Treatment + Tissue + Treatment x Tissue + Residual. Prior to analysis all data was tested for homogeneity of variance using Cochran’s test. Any data failing to meet this assumption was transformed. If the transformed data still did not meet the assumptions, analysis was still undertaken as balanced multifactorial ANOVAs with a large n (>5) are robust for departures from these assumptions [29]. Where interactions or main terms were significant, post hoc Student-Newman-Keuls testing was undertaken. Over the course of the experiment there was successive mortality in the bleached and recovering corals. As such, to keep the design balance and to allow pairing of genotypes, those genotypes that experienced mortality at a given recovery interval were removed from the analysis. No multiple test corrections, such as Bonferroni corrections, were applied. Although multiple ANOVAs have been undertaken, each test is examining an independent hypothesis and as such, corrections to reduce type 1 error are not appropriate, and these corrections increases the chances of type 2 errors [30].

To illustrate the relative levels of photoautotrophic or heterotrophic enrichment between the species and recovery intervals, the mean and standard error for the difference between the control and the bleached corals for each tissue fraction (i.e., coral host, algal endosymbiont, skeleton) and each genotype of coral for the first 24 hours following incubation in the isotopically labelled environment at each recovery interval were calculated and plotted. This was done by subtracting the individual isotopic values for the control corals from that of the bleached corals for each tissue fraction from each fragment within each genotype pair. This was then averaged over the 4, 12, and 24 hour chase intervals for each tissue fraction from each coral fragment. Next the average difference for each fraction within each treatment and species was calculated along with its corresponding standard error. This was repeated for each recovery interval (i.e., 0, 1, 4, and 11 months). As such, this highlights the relative difference between the bleached and non-bleached tissue for each tissue fraction, at each recovery interval and for each type of pulse-chase. In doing so the magnitude of any differences between the tissue fragment types is lost. 

## Results

### Experimental Conditions

**Figure 2:**
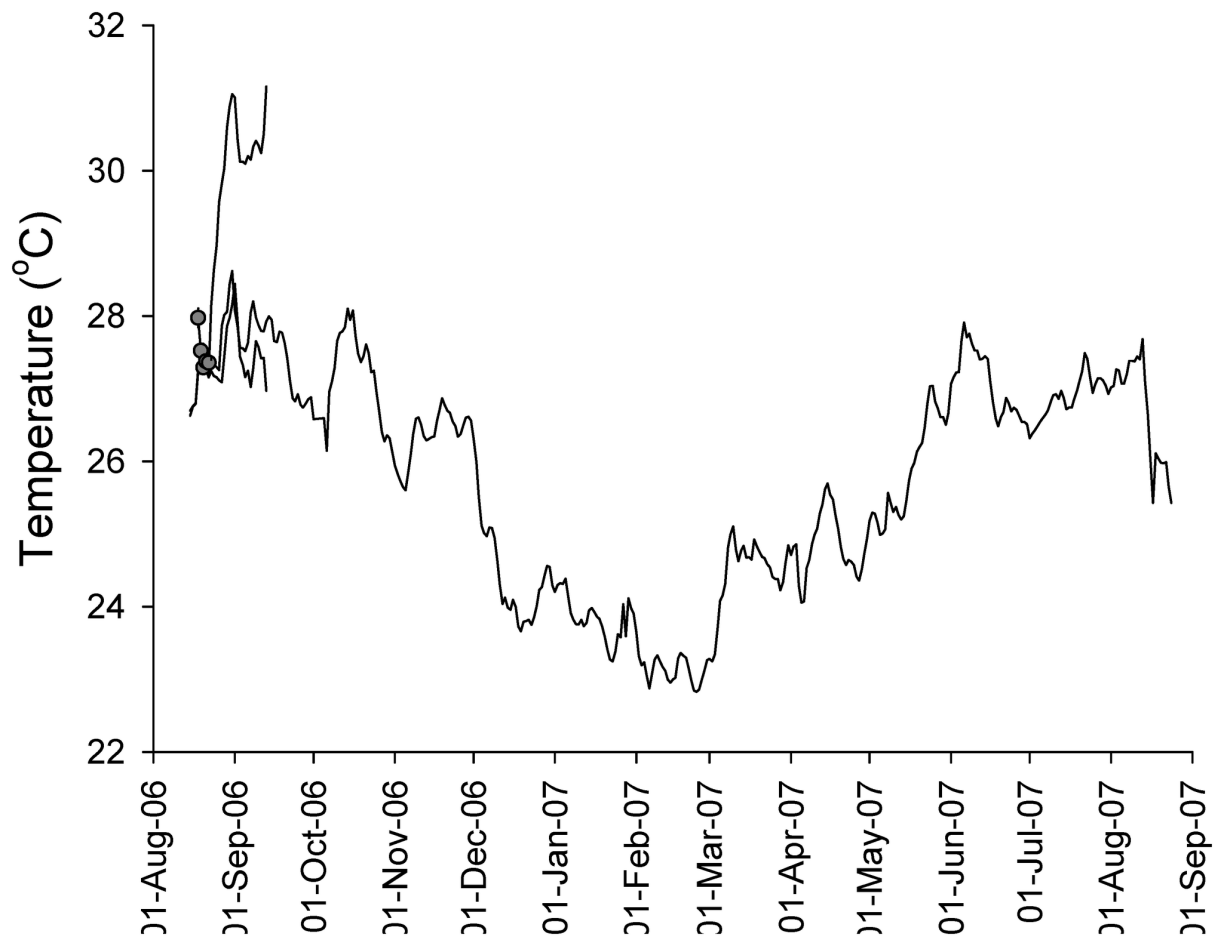
Average daily temperatures on the reef (grey), in the non-bleached control tanks (black), and in the treatment tanks (white). Error bars are the same size or smaller than the symbols.

**Figure 3:**
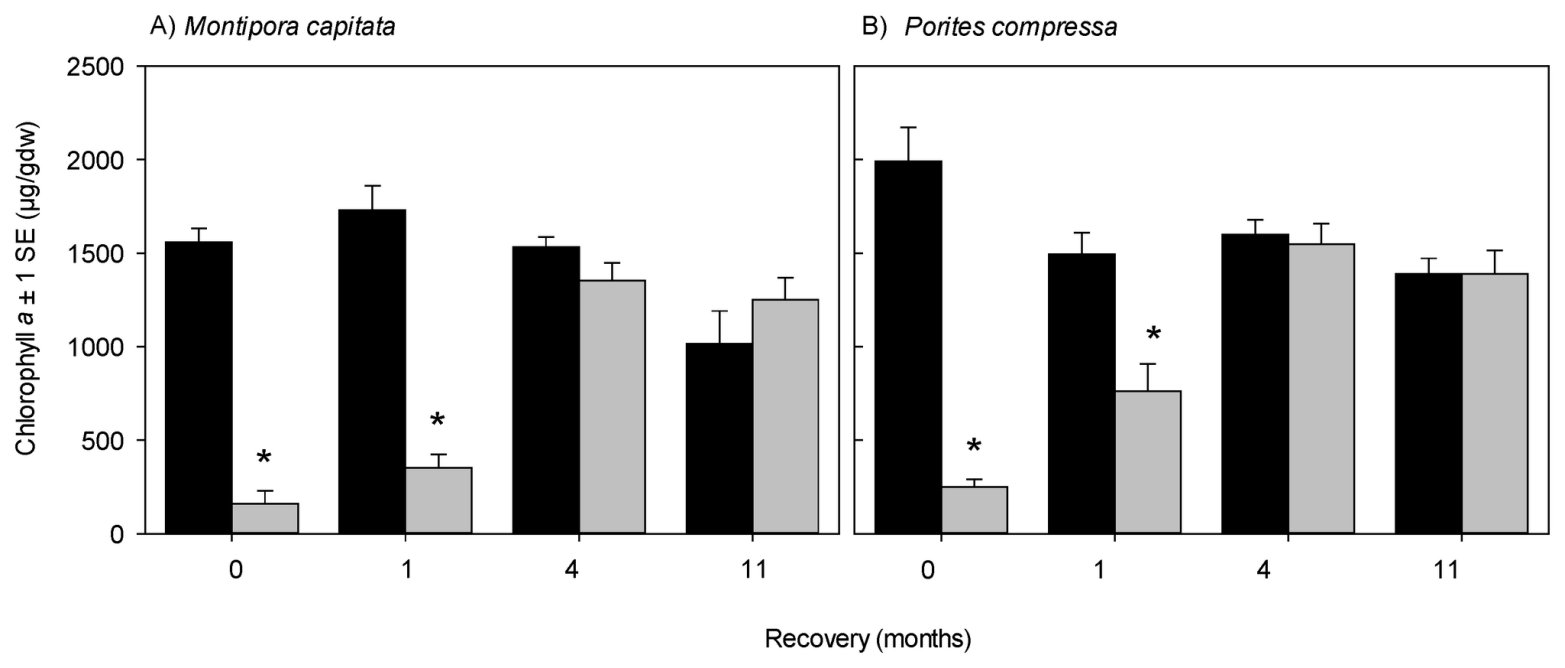
Mean chlorophyll *a* contents (± 1 standard error (SE)) of non-bleached control (black) and bleached (gray) A) *Montipora capitata* and B) *Porites compressa* corals. Within each species, statistically significant differences between non-bleached control and bleached corals at each recovery interval are indicated with an *. Results of ANOVA statistics given in Table 1.

The average water temperature of the tanks over the course of the experiment was 30.2 °C (**±**0.20 SE) for the bleached coral tanks, and 27.4 °C (**±**0.08 SE) for the non-bleached coral tanks (Figure 2). At the end of 3.5 weeks in the tanks the corals were visibly bleached (white in colour) and average chlorophyll *a* (Chl*a*) values of the bleached corals were significantly lower than that of the non-bleached corals (Table 1, Figure 3) such that bleached *M. capitata* and *P. compressa* had only 10.3% and 7.6% of the Chl*a* of their non-bleached counterparts, respectively. This significant difference was maintained through the first month of recovery at which stage the corals were still visibly bleached. However after 4 months, the Chl*a* had recovered in the bleached corals such that there was no longer a significant difference compared to the control. This state persisted until the end of the experiment, equivalent to 11 months of recovery, where again there was no significant difference in Chl*a* between the treatments.

**Table 1:** *Montipora capitata* and *Porites compressa* Chl a concentrations.

Source	*Montipora capitata*				*Porites compressa*			
Recovery T=0	DF	SS	F	P	DF	SS	F	P
Treatment	1	10252965	188.54	<0.001	1	26174978	34.47	<0.001
Residual	19	1033234			41	31133390		
Total	20	11286199			42	57308368		
Recovery T=1								
Treatment	1	9481689	86.62	<0.001	1	2683013	15.59	0.001
Residual	18	1970404			18	3098363		
Total	19	11452093			19	5781376		
Recovery T=4								
Treatment	1	161530	2.68	0.119	1	13497	0.15	0.706
Residual	18	1085122			18	1649368		
Total	19	1246652			19	1662865		
Recovery T=11								
Treatment	1	276678	1.24	0.28	1	3	0.001	0.996
Residual	18	4009815			17	1794724		
Total	19	4286493			18	1794727		

Analysis of variance (ANOVA) of the Chl *a* concentrations for bleached and non-bleached control corals (DW = dry weight) at each recovery interval.

### Photoautotrophically Acquired Carbon

**Figure 4:**
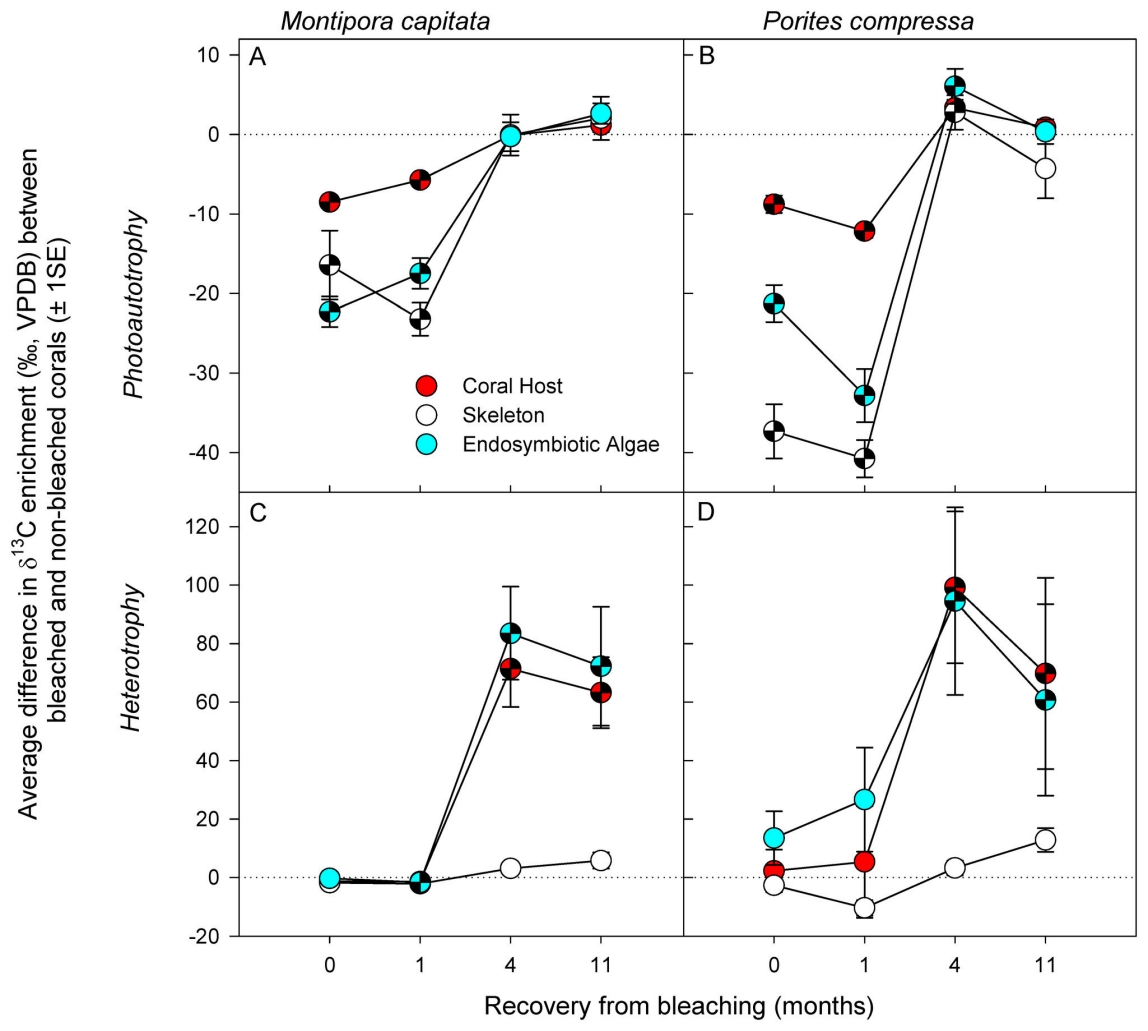
Relative assimilation of ^13^C-labelled carbon via photoautotrophy (panels A, B) and heterotrophy (panels C, D) in *Montipora* capitata (A, C) and *Porites compressa* (B, D) corals. Values represent the average of the difference between the enrichment values of the control and the bleached corals for each genotype and for each tissue type (coral host = red; skeleton = white; endosymbiotic algae = blue). The dashed line represents no difference between the control and the bleached corals in terms of their total isotopic enrichment. Chequered symbols below the dashed line indicate that carbon assimilation by bleached corals was significantly less than that of non-bleached control corals. Chequered symbols above the dashed zero line indicate that carbon assimilation by bleached corals was significantly in excess of that by non-bleached control corals.

Immediately after bleaching (zero months recovery) and after 1 month of recovery, photoautotrophic carbon assimilation was significantly lower in all three coral components (the coral host, endosymbiotic algae, and skeleton) of bleached compared to non-bleached control in the *M. capitata* corals (Figure 4A, Table 2) and in *P. compressa* (Figure 4B, Table 3). After four months of recovery, there was no longer a significant difference in the carbon assimilation for any of the three coral components for *M. capitata* between the bleached and non-bleached corals (Figure 4A). This is in contrast to *P. compressa* which showed than the bleached corals (all three components) had assimilated significantly more carbon that the control corals (Figure 4B). By 11 months of recovery, there were no significant differences in the assimilation of photoautotrophic carbon between bleached and non-bleached control corals for either species. The average isotopic enrichment for bleached corals was 10.8 (±0.6 s.e.) and was 22.3 (±0.8 s.e.)‰VPDB for non-bleached corals.

**Table 2:** *Montipora capitata* photoautotrophic label.

Recovery	0				1				4				11			
Number	N=15,				N=15,				N=11				N=9			
Transformation	lnx+3				None				None				None			
Cochrans Test	C=0.31, P>0.05				C=0.34 P<0.05				C=0.43, P<0.01				C=0.40, P<0.05			
Source	SS	DF	F	P	SS	DF	F	P	SS	DF	F	P	SS	DF	F	P
Treatment(Tr)	37.81	1	128.26	<0.01	5385.71	1	206.06	<0.01	0.83	1	0.03	0.87	51.34	1	1.63	0.21
Tissue (Ti)	14.46	2	24.53	<0.01	2802.73	2	53.62	<0.01	3068.54	2	52.49	<0.01	1523.55	2	24.13	<0.01
Tr x Ti	1.89	2	3.2	0.046	1192.92	2	22.82	<0.01	0.11	2	0	0.99	4.78	2	0.08	0.93
Residual	24.77	84			2195.48	84			1753.65	60			1515.12	48		
Total	78.93	89			11576.83	89			4823.13	65			3094.80	53		

Analysis of variance (ANOVA) of the δ^13^C enrichment following an 8h incubation with DI-^13^C-labeled seawater. Post hoc Student- Newman-Keuls (SNK) tests were used when terms were significant. Tr = treatment (bleached, non-bleached control); Ti = tissue type (coral host, endosymbiotic algae, skeleton).

**Table 3:** *Porites compressa* photoautotrophic label.

Recovery	0				1				4				11			
Number	N=15				N=13				N=11				N=9			
Transformation	ln(x+3)				None				None				None			
Cochrans Test	C=0.31 NS				C=0.40 P<0.01				C=0.40, P<0.05				C=0.34, NS			
Source	SS	DF	F	P	SS	DF	F	P	SS	DF	F	P	SS	DF	F	P
Treatment(Tr)	40.5	1	304.18	<0.01	15921	1	400.29	<0.01	271.76	1	5.34	0.02	13.902	1	0.38	0.54
Tissue (Ti)	9.92	2	37.22	<0.01	5053.9	2	63.53	<0.01	3910.9	2	38.4	<0.01	1920.9	2	26.3	<0.01
Tr x Ti	2.16	2	8.09	<0.01	2831.23	2	35.59	<0.01	33.947	2	0.33	0.72	73.017	2	1	0.38
Residual	11.2	84			2863.68	72			3053.1	60			1755.7	48		
Total	63.8	89			26669.8	77			7269.7	65			3763.4	53		

Analysis of variance (ANOVA) of the δ^13^C enrichment following an 8 h incubation with DI-^13^C-labeled seawater. Post hoc Student- Newman-Keuls (SNK) tests were used when terms were significant. Tr = treatment (bleached, non-bleached control); Ti = tissue type (coral host, endosymbiotic algae, skeleton).

### Heterotrophic Labelling

Immediately after bleaching (zero months recovery) there was no significant difference in the amount of heterotrophically assimilated carbon between the bleached and non-bleached corals in any of the three components for either species (Figure 4C & D, Table 4 & 5). During the first month of recovery, heterotrophic carbon assimilation in *M. capitata* either did not significantly differ between bleached and non-bleached controls or was slightly (but significantly) lower in bleached than in non-bleached controls. During the same period there was no significant difference in any of the coral components between the bleached and non-bleached fragments of *P. compressa*. However at four months recovery, heterotrophic carbon assimilation by both the coral host and endosymbiotic algae of both species was significantly higher in bleached corals compared to the non-bleached control corals. This extra heterotrophic carbon assimilation was still evident for both species even at the 11 month recovery interval. However, heterotrophic carbon assimilation in the skeletal fraction was no different between bleached and non-bleached control corals of both species at any time during the 11 months of recovery (Figure 4 C & D). The average isotopic enrichment for bleached corals was 59.6 (±4.7s.e.) and was 34.9 (±2.7 s.e.)‰VPDB for non-bleached corals.

**Table 4:** *Montipora capitata* heterotrophic label.

Recovery	0				1				4				11			
Number	N=15				N=14				N=15				N=7			
Transformation	None				Sqrt(X+1)				Ln(X+1)				Sqrt(X+1)			
Cochrans Test	C=0.29, NS				C=0.34, NS				C=0.33				C=0.36			
Source	SS	DF	F	P	SS	DF	F	P	SS	DF	F	P	SS	DF	F	P
Treatment(Tr)	28.1	1	2.06	0.16	2.99	1	16.94	<0.01	5.53	1	22.6	<0.01	92.3	1	26.7	<0.01
Tissue (Ti)	1388	2	50.66	<0.01	18.45	2	52.28	<0.01	104.77	2	214	<0.01	283.2	2	41	<0.01
Tr x Ti	9.01	2	0.33	0.72	0.79	2	2.24	0.11	1.66	2	3.38	0.04	22.5	2	3.26	0.05
Residual	1150	84			13.76	78			20.58	84			124.3	36		
Total	2575	89			35.99	83			132.53	89			522.4	41		

Analysis of variance (ANOVA) of the δ^13^C enrichment following an 11 h incubation with ^13^C labelled rotifers. Post hoc Student-Newman-Keuls (SNK) tests were used when terms were significant. Tr = treatment (bleached, non-bleached control); Ti = tissue type (coral host, endosymbiotic algae, skeleton).

**Table 5:** *Porites compressa* heterotrophic label.

Recovery	0				1				4				11			
Number	N=15				N=12				N=12				N=8			
Transformation	Ln(X+1)				None				Sqrt(X+1)				None			
Cochrans Test	C=0.25, NS				C=0.31, NS				C=0.29, NS				C=0.38, NS			
Source	SS	DF	F	P	SS	DF	F	P	SS	DF	F	P	SS	DF	F	P
Treatment(Tr)	0.3	1	0.40	0.53	939	1	0.56	0.46	132.0	1	24.7	<0.01	27427.7	1.0	6.26	0.02
Tissue (Ti)	59.5	2	35.38	<0.01	85233	2	25.53	<0.01	1043.5	2	97.6	<0.01	116660.5	2.0	13.3	<0.01
Tr x Ti	4.0	2	2.38	0.10	4151	2	1.24	0.30	51.0	2	4.77	0.01	7495.9	2.0	0.86	0.43
Residual	70.7	84			110181	66			352.9	66			184044.4	42.0		
Total	134.5	89			200504	71			1579.4	71			335628.5	47.0		

Analysis of variance (ANOVA) of the δ^13^C enrichment following an 11h incubation with ^13^C labelled rotifers. Post hoc Student-Newman-Keuls (SNK) tests were used when terms were significant. Tr = treatment (bleached, non-bleached control); Ti = tissue type (coral host, endosymbiotic algae, skeleton).

## Discussion

Understanding how corals respond to, and recover from, bleaching events is crucial if we are to better predict the impacts of global warming on coral reef ecosystems. Our data show that the recovery pattern of the trophic behaviour of the coral holobiont is complex and non-uniform. Although the photoautotrophic mechanism had recovered after 4 months, with Chl*a* and photoautotrophic carbon assimilation levels the same between bleached and non-bleached corals of both species, the assimilation of heterotrophic carbon was highly elevated in the bleached corals compared to the non-bleached controls even after 11 months of recovery. The failure of heterotrophic carbon assimilation to return to non-bleached levels even after 11 months of recovery suggests that either 1) bleaching induces an acclimatization response that could buffer them from future bleaching, or 2) the temporal refugia for corals from bleaching events is greater than 11 months long. We explore these findings in more detail below.

The photoautotrophic system of both species was still in recovery after 1 month as demonstrated by the lower levels of carbon assimilation and Chl*a* in bleached relative to the non-bleached corals. This is consistent with previous observations of coral bleaching reducing photosynthetic rates in these species by 67-90% [9] and also reduced CZAR (contribution of zooxanthellae-acquired carbon to daily animal respiration) by approximately 60% in these species [21,31]. Associated with the impact on the photoautotrophic system there was a lower assimilation of carbon into the skeletal component in the bleached corals relative to the non-bleached, which is also consistent with previous studies that have shown a reduction or cessation of skeletal growth as a result of bleaching in these species [26,32,33]. 

After 4 months of recovery, both Chl*a* and photoautotrophic carbon assimilation rates indicated that photoautotrophy had fully recovered in *M. capitata*. At the same time, *P. compressa* had recovered Chl*a* and was assimilating significantly more photoautotrophically derived carbon than the controls. This may be due to *P. compressa* not increasing its feeding rates when bleached [21,31], and thus relying predominantly on photosynthesis to promote recovery. By 11 months, there were no significant differences in the assimilation of photoautotrophic carbon between bleached and non-bleached control corals for either species. Thus for these two coral species, photoautotrophy had recovered within 4 months of bleaching. These findings show that bleached corals had visibly recovered and photosynthetic pigment concentrations and photoautotrophic carbon assimilation were at normal or higher levels after only 4 months of recovery. This corresponds to field estimates of the duration of coral recovery based on appearance, pigment concentration, and photosynthetic activity which range from between 25 days to over 11 months [9,34-36]. 

While photoautotrophic carbon is clearly important for recovering corals, heterotrophic carbon specifically appears to be critical for the survival of corals during long-term recovery, and consequently may be the variable that defines the extent of the temporal refugia. During the first month of recovery, heterotrophic carbon assimilation in *M. capitata* either did not significantly differ between bleached and non-bleached controls or was slightly lower in bleached than in non-bleached controls. Yet, previous work has clearly shown that feeding rates of *M. capitata* dramatically increase following bleaching [21,31]. Thus, the extra heterotrophic carbon acquired by *M. capitata* in the early stages of recovery is not being assimilated but is being rapidly catabolized to meet metabolic demand and/or is lost via mucus or particulate organic matter. This is consistent with findings from bleached Hawaiian *Porites lobata* corals that also catabolize their heterotrophically acquired carbon [37] and findings by Tanaka et al (2009) showing that bleached corals lost heterotrophically acquired carbon through mucous production or as particulate carbon. In addition, preliminary measurements of dissolved organic carbon (DOC) fluxes in *M. capitata* suggests that it also releases DOC when bleached (Hughes & Grottoli unpublished). However further experimental work is required to test this. For *P. compressa*, the lack of a significant difference in heterotrophic carbon assimilation after 1 month of recovery is consistent with a lack of any changes in feeding rates in this species with bleaching [21,31].

However after 4 months of recovery, heterotrophic carbon assimilation by both the coral host and endosymbiotic algae of both species was dramatically higher in bleached corals compared to the non-bleached control corals. The trigger for this increase in heterotrophic carbon assimilation is unknown, but bleaching depletes specific lipid classes [38] and the physiological change may elicit this response. This extra heterotrophic carbon assimilation was still evident for both species even after 11 months of recovery for which there are two possible interpretations. Firstly, it has been previously shown that for these species the tissue biomass, lipid, protein and carbohydrate recovers within 8 months post bleaching [9]. This, combined with the evidence presented here that the photoautotrophic system had recovered within 4 months, suggests that increased heterotrophic assimilation during long-term recovery is an adaptive response that enhances production through heterotrophy which, could increase coral resilience to future bleaching events. Previous experiments have also shown that heterotrophic carbon is the carbon source for tissue building in corals and anemones [4,24] and that heterotrophic feeding by corals can diminish the severity of bleaching [25]. This hypothesis is also consistent with model scenarios predicting that heterotrophy may be an important determinant of colony survival after bleaching [22]. Alternatively, this heterotrophic compensation is evidence that the corals are still in recovery after 11 months despite the recovery of other physiological parameters. Optimal foraging theory [39] suggests that if the capacity to increase heterotrophic carbon assimilation was beneficial to non-bleached corals, then there would be no difference between the bleached and non-bleached corals. As the heterotrophic compensation was only observed in the bleached corals, it supports the interpretation that it is a direct response to the bleaching stress and is part of the recovery process. This is the first definition of the temporal refugia for a coral species based on these measurements and is considerably longer than previous estimates based on growth rates or reproductive tissue [26,33,40], suggesting that full recovery can take significantly longer than previously thought and that the temporal refugia from climate change required is greater than originally assumed.

The response to bleaching events varies between species and within individuals of the same species [41,42]. This in part due to past thermal history whereby those corals having previously experienced thermal stress are less susceptible to future bleaching [43] and also in part due to the ability of some corals to adapt or acclimatise to thermal stress. The mechanisms of this adaption are poorly defined. Adaptive change by the holobiont to coral bleaching has been previously observed through the ability of the coral holobiont to shuffle or switch the endosymbiotic algae it houses, switching from less thermally tolerant clades to more tolerant clades following bleaching [44,45]. Another possible mechanism is a high degree of physiological plasticity in the relationship between the host and the endosymbiotic algae such as the up regulation of heat shock proteins [46] allowing a more stable relationship between the coral animal and endosymbiotic algae during thermal stress. In addition, heterotrophic plasticity has been shown to maintain physiological status in corals immediately following bleaching [21]. At a community scale, natural selection on ecological timescales has also been posited as a mechanism of adaption. Here we show for the first time that heterotrophic compensation persists for almost a year following bleaching, highlighting the long-term importance of heterotrophic carbon in coral physiology for 11 months after bleaching. The increased heterotrophic carbon assimilation following bleaching may 1) act as an adaptive strategy against further bleaching events by increasing the nutrient acquisition through heterotrophy and possibly reducing the dependence of the holobiont on the photoautotrophic system (however further experimentation is required to test this hypothesis) or 2) be a symptom of a coral still suffering negative effects of bleaching and for whom the size of the temporal refugia required is greater than 11 months. 

In light of these findings long-term recovery from bleaching is critically dependent on healthy plankton populations throughout the year. Healthy coral reefs are known to have a concomitant and dramatic impact on plankton populations in overlying waters, depleting pelagic diatoms and zooplankton by as much as 90% and 60%, respectively [47]. Increasing sea surface temperatures in the tropics over the past few decades have resulted in a steady decline in zooplankton abundance [48] with marked decreases in plankton during bleaching events on reefs [49]. In the future, a potentially chronic need for extra heterotrophic carbon by corals due to multiple and possibly annual bleaching events, combined with decreases in zooplankton populations due to warming, would ultimately limit the quantity and quality of plankton available on reefs needed to support recovery from bleaching and to build future resilience to repeated bleaching events. 
